# PLAC8 is an innovative biomarker for immunotherapy participating in remodeling the immune microenvironment of renal clear cell carcinoma

**DOI:** 10.3389/fonc.2023.1207551

**Published:** 2023-10-30

**Authors:** Xu Sun, Zhanpeng Liu, Qian Yu, Yinwei Chen, Yunwen Sun, Qingyi Zhu, Jian Yang, Rongjiang Jiang

**Affiliations:** ^1^ Department of Urology, The Second Affiliated Hospital of Nanjing Medical University, Nanjing, China; ^2^ Department of Urology, The First Affiliated Hospital of Nanjing Medical University, Nanjing, China; ^3^ School of Pediatrics, Nanjing Medical University, Nanjing, China; ^4^ Department of Urology, Jinhua Municipal Central Hospital, Jinhua, China; ^5^ The First Clinical Medical College of Nanjing Medical University, Nanjing, China

**Keywords:** PLAC8, ccRCC, immune microenvironment, prognosis, biomarker

## Abstract

**Background:**

PLAC8 has been identified in the progression of various cancers by inducing tumorigenesis, immune response, chemotherapy resistance and metastasis. Nevertheless, the precise biological function of PLAC8 in renal cancer remains unknown.

**Methods:**

We obtained the expression profile and associated clinical characteristics of patients diagnosed with clear cell renal cell carcinoma (ccRCC) from The Cancer Genome Atlas database. The biological behavior of specific cell lines was detected using Cell Counting Kit-8 (CCK-8), colony formation, and 5-ethynyl-2’-deoxyuridine (EdU) assay. A prognostic model was constructed based on PLAC8-related molecules through a machine-learning algorithm.

**Results:**

We observed overexpression of PLAC8 in ccRCC patients. In addition, PLAC8 has been identified as being linked to unfavorable clinical characteristics and adverse prognosis outcomes. Biological enrichment analysis revealed the potential involvement of PLAC8 in cell cycle checkpoints, mitotic phase transformation, immunotherapy-predicted and reactive oxygen species (ROS) related pathways. In addition, immune analyses showed that PLAC8 was involved in remodeling the tumor microenvironment (TME) and affecting the effect of immunotherapy in ccRCC patients. *In vitro* experiments demonstrated a significant reduction in the proliferation, invasion and migration of renal cancer cells following the knockdown of PLAC8. Finally, LASSO logistics regression was applied to construct a prognosis model, which presented a favorable prediction ability on the prognosis of ccRCC.

**Conclusion:**

Our results implied that PLAC8 may be a novel immunotherapy biomarker of ccRCC, which is a crucial molecule in remodeling the cancer microenvironment. PLAC8 can predict immunotherapy response and is expected to guide precise treatment.

## Introduction

Renal cell carcinoma (RCC) arises from the renal parenchymal urinary tubular epithelial cells and represents a highly lethal urogenital malignancy. Its mortality rate ranges from 30% to 40% in contrast to bladder and prostate cancers (1). With the changes in lifestyle and the rise of obesity, hypertension, and other diseases, the incidence of RCC has increased by about 2% annually over the past 20 years. In China, RCC ranks second in urinary system tumors, burdening socio-economic development heavily (2, 3). ccRCC can be cured through surgery or ablative methods in its early stages, nearly one-third of patients exhibit distant metastases upon initial diagnosis. These unfortunate circumstances contribute to a noteworthy decline in the 5-year survival rate, which stands at a mere 32% for individuals with advanced-stage ccRCC (3). Given the immunogenic nature of ccRCC, immune checkpoint inhibitor (ICI)-based therapies with programmed cell death 1 (PD-1), programmed cell death one ligand 1 (PD-L1) or cytotoxic T lymphocyte antigen 4 (CTLA-4) pathway blockers are increasingly dominant as the current frontline treatment for metastatic disease (4). However, ccRCC is a heterogeneous disease, and its immunotherapy can produce diverse clinical outcomes even in patients with similar clinical features (5). Therefore, it is of great significance to search for effective prognostic signatures for accurately predicting the response to immunotherapy.

With the advent of the genomic era, emerging bioinformatics analysis has allowed researchers to explore specific molecular mechanisms involved in disease development more deeply and conveniently. PLAC8 has been implicated in the progression of multiple cancer types, including breast, lung, prostate, and colon cancer, by inducing tumorigenesis, immune response, chemotherapy resistance (6). Jia Y et al. indicated the novel KLF4/PLAC8 signaling pathway in the malignant progression of lung cancer by regulating tumor cell proliferation and apoptosis (7). Li C et al. demonstrated that colon cancer PLAC8-overexpressing cells promoted unconventional epithelial-to-mesenchymal transition dependent on increased phosphorylated extracellular signal-regulated kinase 2 (8). However, no previous study focused on the potential function of PLAC8 in the immunotherapy of patients with ccRCC.

In this study, PLAC8 overexpression in ccRCC patients was shown to be associated with worse clinical parameters and poorer overall survival. The biological role of PLAC8 in ccRCC was explored by enrichment analysis. In addition, we investigated the contribution of PLAC8 in reshaping the immune microenvironment of ccRCC and its implications for immunotherapy efficacy. By knocking down PLAC8 *in vitro*, it was found that the proliferation, invasion and migration of RCC cells were significantly reduced. Finally, the prognostic model constructed by PLAC8-derived molecules has a strong predictive ability for the prognosis of patients.

## Methods

### Data acquisition and processing

We obtained the expression profile and related clinical information of ccRCC patients from The Cancer Genome Atlas (TCGA) database, accessible at https://portal.gdc.cancer.gov/ (9). Tumor mRNA expression data was obtained from the UCSC Xena website (https://xenabrowser.net/) for further analysis. Data preprocessing was executed in the R environment, employing the limma and affy packages for the required procedures. Immunohistochemical images of renal cancer were obtained from the Human Protein Atlas (HPA) database (10). The nomogram was created using the survival and RMS software and evaluated through calibration curves and Decision Curve Analysis (DCA). The baseline information of included patients was shown in **Table 1**.

**Table 1:** Clinicopathological characteristics of KIRC patients.

Variables	Type	Cases	Percentage(%)
Tissue	Normal	71	12.2
	Tumor	513	87.8
Age(years)	<70	387	66.2
	≥70	126	21.6
	Unknown	71	12.2
Gender	Female	176	30.1
	Male	337	57.7
	Unknown	71	12.2
Race	White	443	75.9
	Black	55	9.4
	Unknown	86	14.7
Grade	G1-2	228	39.0
	G3-4	272	46.6
	Unknown	84	14.4
Stage	Stage I-II	307	52.6
	Stage III-IV	203	34.7
	Unknown	74	12.7
T stage	T1-2	325	55.6
	T3-4	188	32.2
	Tx	71	12.2
M stage	M0	405	69.3
	M1	77	13.2
	Mx	102	17.5
N stage	N0	227	38.9
	N1	16	2.7
	Nx	341	58.4
Laterality	Left	237	40.6
	Right	275	47.1
	Unknown	72	12.3

### Biological enrichment

ClueGO, a Cytoscape Application, identifies and visualizes the Gene Ontology terms for the input molecules, presenting them as molecular interaction networks (11). The Gene Ontology analysis was conducted utilizing the clusterProfiler package within the R environment (12, 13). Employing Gene Set Enrichment Analysis (GSEA), we analyzed the signaling pathways of the hub genes and potential biological variations (14, 15). Quantitative enrichment of scores based on specific reference documents was performed through single sample GSEA (ssGSEA).

### Analysis of immune cells and immune infiltration

Multiple algorithms were utilized to quantify the immune microenvironment, including the TIMER, EPIC, MCPCOUNTER and QUANTISEQ algorithms (16–18). By analyzing the transcriptional profile, we employed the Immunophenoscore (IPS) to evaluate the immunotherapeutic role of the IPS score in ccRCC patients (19). Meanwhile, we evaluated immunotherapy response in patients with ccRCC using the Tumor Immune Dysfunction and Exclusion (TIDE) algorithm (20).

### Genomic characterization

We retrieved two potential predictive biomarkers for immunotherapy, including the tumor mutation burden (TMB) and microsatellite instability (MSI) score, from the TCGA database. The gene mutation characteristics of PLAC8 were obtained and visualized from the TCGA database via the online website https://www.home-for-researchers.com/.

### Construction of the prognosis model based on machine learning

Random allocation was used to divide all patients into training and internal validation in a ratio of 2:1. We utilized the E-MTAB-1980 project as the external validation cohort. We conducted differential expression analysis of genes (DEGs) to compare patients with high and low PLAC8 expression. Univariate Cox regression analysis aimed to identify molecules significantly associated with patient clinical outcomes. We utilized LASSO logistic regression to optimize variables, which were subsequently used as inputs for further multivariate Cox regression analysis (21). Through our analysis, we successfully identified a prognosis signature characterized by the formula: “Risk score = Expression of A * Coef A + Expression of B * Coef B + … + Expression of X * Coef X”

### Cell lines, qPCR, retroviral infection, and transfection

The National Collection of Authenticated Cell Cultures (Shanghai, China) provided a normal human kidney cell line (HK-2) and RCC cell lines (786-O, Caki-1, Caki-2, and ACHN) that were acquired The extraction of total RNA from the cell lines was carried out utilizing Trizol reagent. SYBR Green assay was used for qPCR for the analysis of PLAC8 mRNA expression following the manufacturer’s instructions. The primers used were: PLAC8, forward: 5’-GGAACAAGCGTCGCAATGAG-3’; PLAC8, reverse: 5’-AAAGTACGCATGGCTCTCCTT-3’; GAPDH, forward: 5’- CGGATTTGGTCGTATTGGG-3’; GAPDH, reverse: 5’-CTGGAAGATGGTGATGGGATT-3’. We purchased control and knockdown shRNA of PLAC8 from Hanbio. Cell transfection was conducted by lipofectamine 3000 according to the manufacturer’s protocol. Lentivirus-transinfected cells were successfully constructed with puromycin (MCE.NJ). Then we harvested the PLAC8 stable-knockdown cells.

### CCK8 assay

Cell viability was evaluated using the Cell Counting Kit-8 (CCK-8, Dojindo, Shanghai, China). The cells were distributed into 96-well plates at a density of 2000 cells per well. After a two-hour incubation period, ten microliters of CCK8 reagent were introduced to each well (set as 0 hour time node). At the time nodes of 0, 24, 48 and 72 hours, microplate readers measuring absorbance at 450 nm were used to measure the cells’ absorbance.

### Clonogenic assay

At a density of 500 cells per well, the cells were inoculated into a six-well plate. 2 ml growth medium was first added to each plate and replaced every 4 days. Subsequently, the cells were stained with 10% crystal violet dye for a duration of 12 days and counted using a microscope.

### 5-ethynyl-2’-deoxyuridine assay

To analyze cancer cell proliferation, EdU staining was conducted using an EDU kit (RiboBio, China) following the manufacturer’s protocol. Cells were inoculated at a density of 4×10^5^ cells per well into a six-plate well. The cells were initially fixed overnight with 4% formaldehyde. Following that, the cells were washed, neutralized with glycine, and treated with 0.5% TritonX-100 for 30 minutes. Cells were then treated with Apollo® reaction solution for 30 minutes. Image J software was used to count the EdU-positive cells under a fluorescent microscope (Leica) by staining the nuclei with Hoechst 33342.

### Transwell assay

For the Transwell assay, chambers (8 μm core, Corning, USA) were utilized. Matrigel coating was applied to the chambers for infiltration analysis, while chambers without Matrigel were used for migration assessment. Serum-free cell culture media was inoculated into the upper chamber and media enriched with 20% of serum was inoculated into the lower chamber of the Transwell. Cells were inoculated into the upper compartment at a rate of 4×10^4^. Following a 16-hour incubation period, the cells located beneath the membrane were treated with 4% formaldehyde and subsequently stained with crystal violet.

### Statistical analysis

The R software was employed for all procedures conducted in this study. Statistical significance was determined using a threshold of P<0.05. Appropriate statistical methods are selected for different distribution forms of data.

## Results

### Pan-cancer analysis and clinical significance of PLAC8

**Figure 1:**
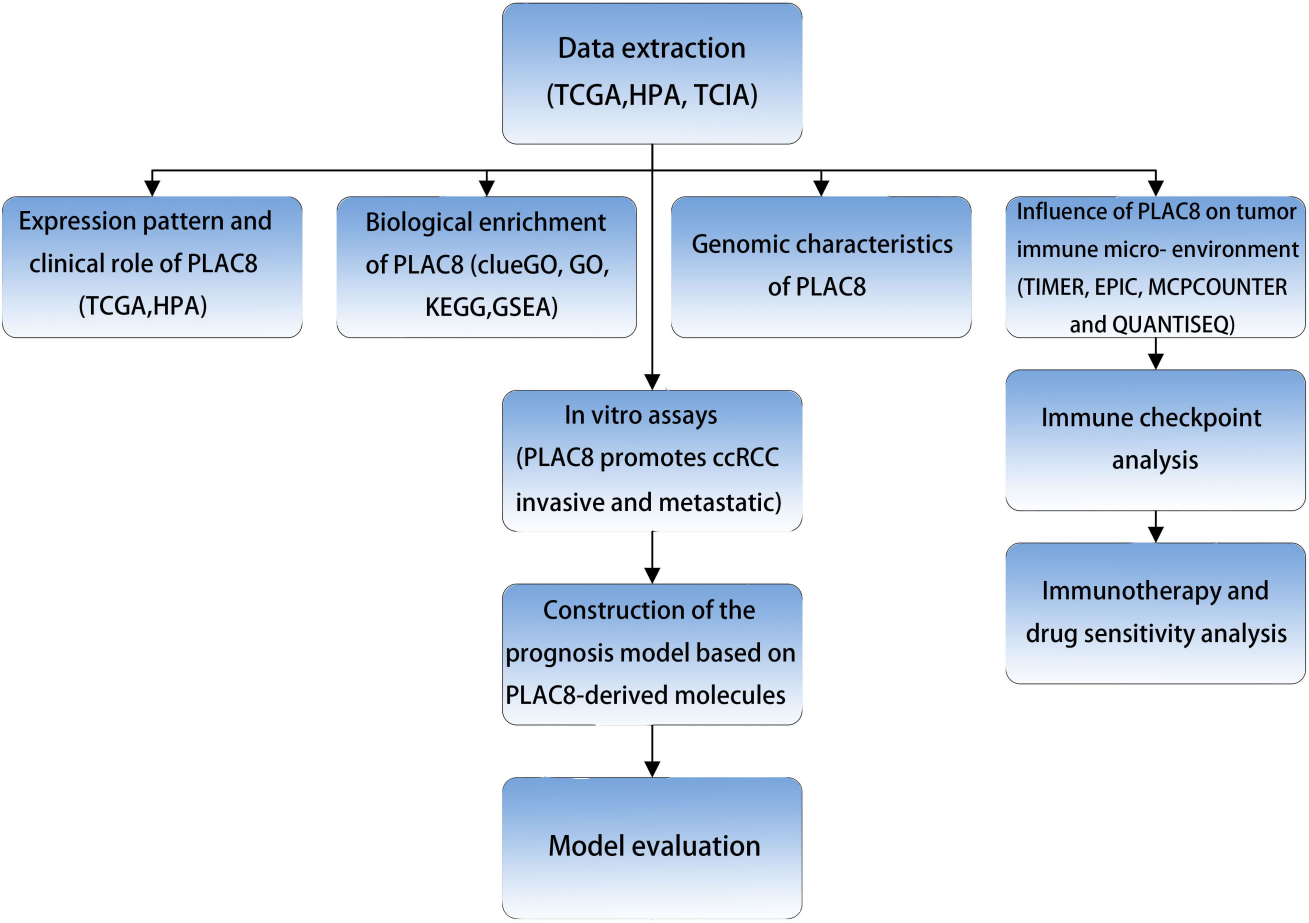
The flow chart of the whole research.

**Figure 2:**
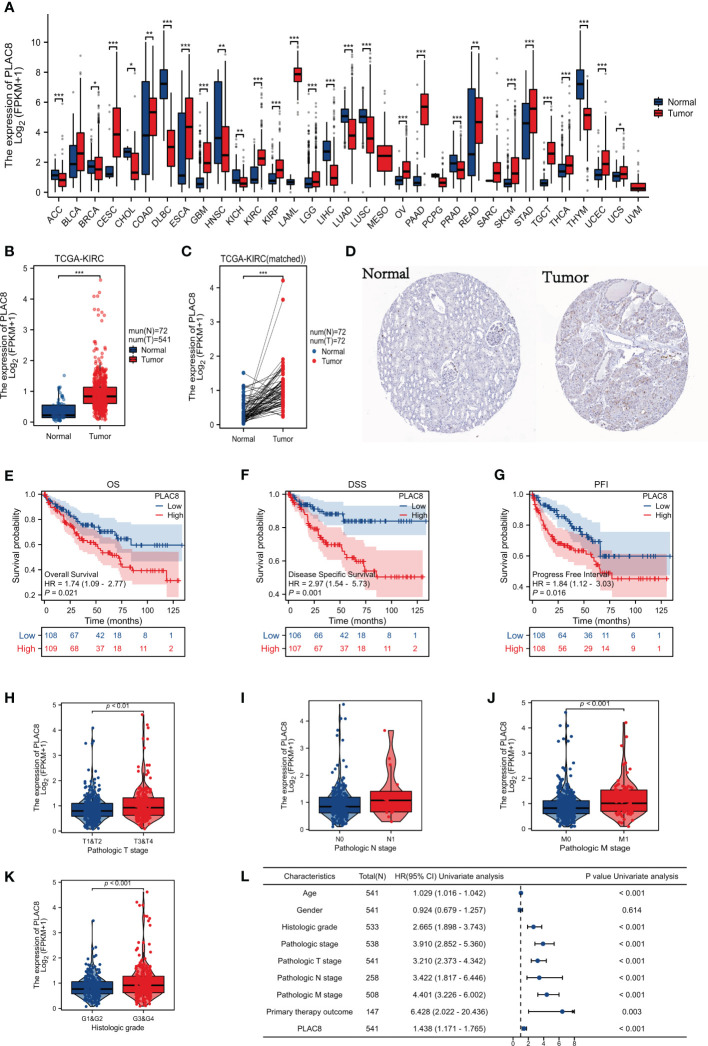
Gene expression and clinical significance of PLAC8 in ccRCC. **(A)** Expression of PLAC8 in pan-cancer; **(B, C)** Expression level of PLAC8 in paired or unpaired samples; **(D)** Immunohistochemical picture (Antibody HPA040465) of PLAC8 obtained from HPA database; **(E-G)** Survival curves of PLAC8 expression in TCGA; **(H-K)** Clinical characteristics analysis of PLAC8; **(L)** Univariate analysis of PLAC8. * = P < 0.05, ** = P< 0.01, *** = P < 0.001.

The procedure of our study is demonstrated in **Figure 1**. Based on the pan-cancer analysis, it was observed that the expression level of PLAC8 was abnormal in most tumor tissues, which may imply its significant role in tumorigenesis (**Figure 2A**). We found that PLAC8 showed different degrees of high expression in ccRCC compared with the normal tissue and matched samples (**Figures 2B, C**). With the help of the HPA database, the immunohistochemical result of the PLAC8 protein level was observed to be higher in ccRCC tissues (**Figure 2D**). Moreover, we attempted to explore the role of PLAC8 in survival and clinical prognosis. Based on the survival curves for OS, DSS, and PFI, it was suggested that higher expression of PLAC8 could potentially be linked to unfavorable prognosis in ccRCC (**Figures 2E-G**). Further, we investigated the corresponding clinical staging indicators. The expression level of PLAC8 was significantly elevated in patients with worsened T-stage, M-stage, and histological grade, while no significant difference was noticed in N-stage (**Figures 2H-K**). The results of the univariate analysis are shown in **Figure 2L**.

### PLAC8 serves as a biological regulator in ccRCC

**Figure 3:**
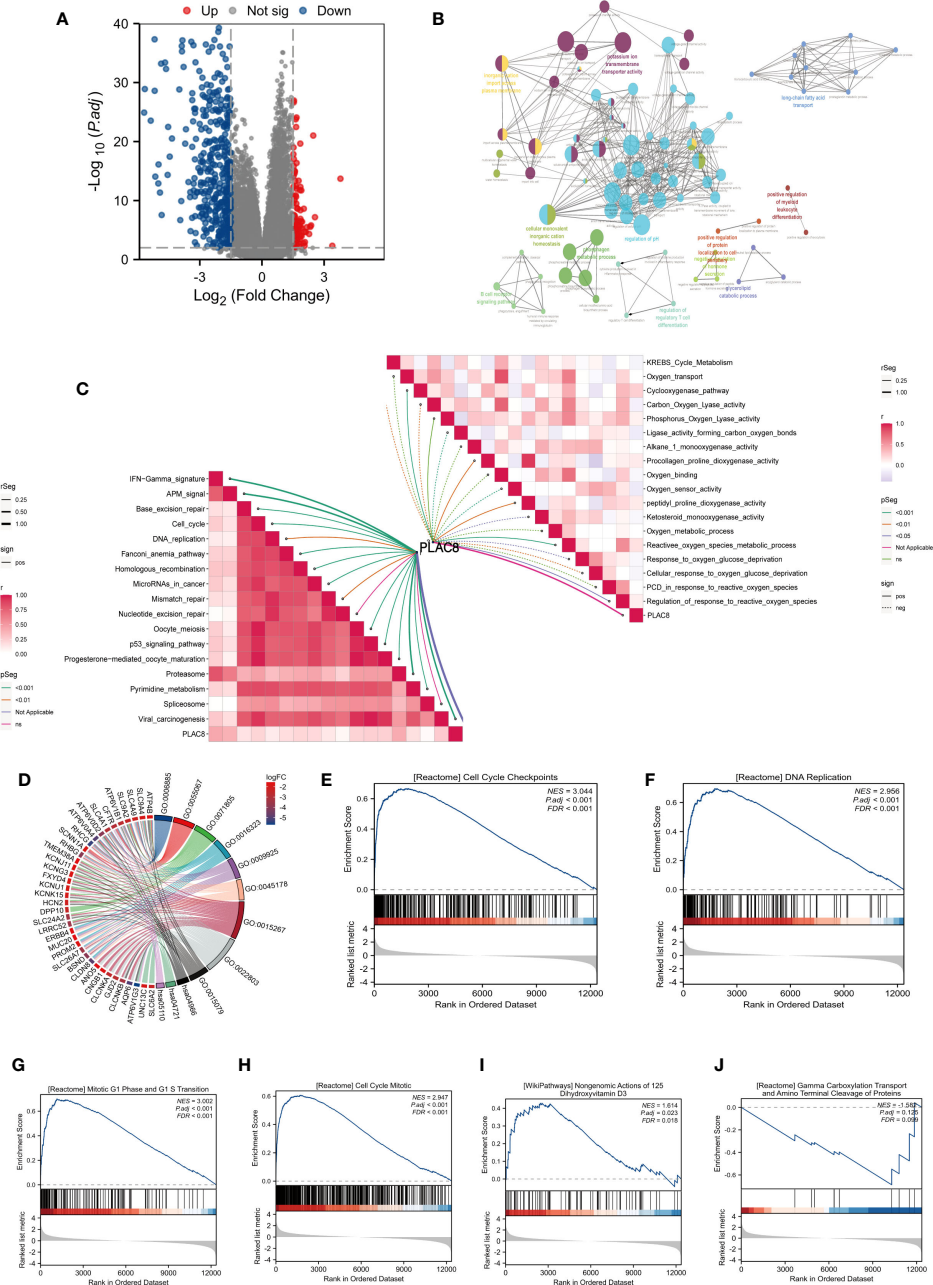
Biological function analysis of PLAC8. **(A)** DEGs between different expression level of PLAC8; **(B)** clueGO analysis based on DEGs; **(C)** Correlation analysis of PLAC8 with immunotherapy-related pathways and reactive oxygen species-involved processes; **(D)** GO analysis of the DEGs; **(E-J)** GSEA enrichment analysis.

We identified 109 upregulated genes and 415 downregulated according to the different expression levels of PLAC8 (**Figure 3A**). The clueGO analysis revealed that the DEGs primarily engage in significant biological processes, including potassium ion transmembrane transporter activity, B cell receptor signaling pathway, regulation of regulatory T cell differentiation, inorganic cation import across the plasma membrane, cellular monovalent inorganic cation homeostasis, and phosphagen metabolic process (**Figure 3B**). Furthermore, the ssGSEA algorithm analysis demonstrated a strong association between PLAC8 and various immunotherapy-predicted pathways, especially IFN-Gamma_signature, APM_signal, proteasome, and Viral_carcinogenesis, indicating a potential significance in immunotherapeutic interventions. Meanwhile, a negative correlation was also observed in many reactive oxygen species-involved biological processes (**Figure 3C**). The GO analysis revealed the top three enriched biological processes as potassium ion transmembrane transport, monovalent inorganic cation homeostasis, and regulation of pH. In terms of cellular components, the enrichment was observed in the basal part of the cell, basal plasma membrane, and basolateral plasma membrane. Additionally, the enriched molecular functions included passive transmembrane transporter activity, channel activity, and potassium ion transmembrane transporter activity (**Figure 3D**). The GSEA analysis revealed the enrichment in cell cycle checkpoints, mitotic G1 phase and G1/S transition, DNA replication, cell cycle mitotic and reactive oxygen species (ROS) related pathways (**Figures 3E–J**).

### PLAC8 participates in reshaping the immune microenvironment of ccRCC

**Figure 4:**
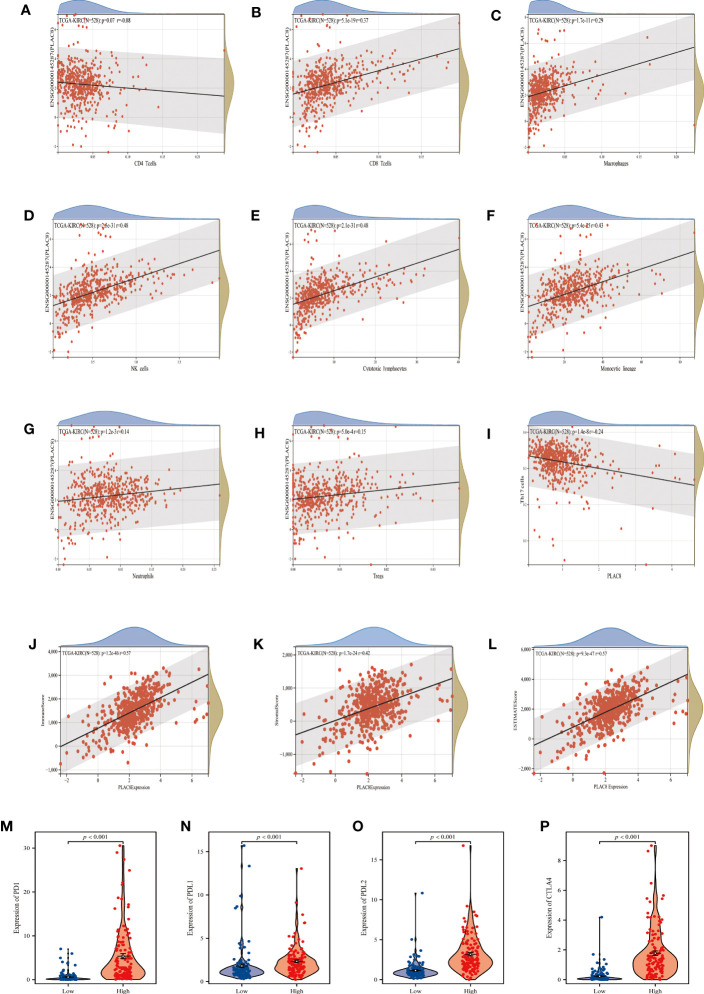
Analysis of immune cells and immune infiltration. **(A–I)** Correlation analysis between PLAC8 and immune cells; **(J–L)** Correlation analysis between PLAC8 and three scores; **(M-P)** Differential expression of common immune checkpoints in different PLAC8 expression group.

By the instrumentality of various immune cell analysis algorithms, including EPIC, MCPCOUNTER and QUANTISEQ, we observed different infiltration patterns in ccRCC patients with diverse expressions of PLAC8. Correlation analysis demonstrated that PLAC8 could increase CD8+ T cells, macrophages, NK cells, cytotoxic lymphocytes, and Monocytic lineage and reduce Th17 cells in the microenvironment of ccRCC (**Figures 4A-I**). Furthermore, a notable positive correlation was identified between PLAC8 and the immune score, stromal score, and estimate score (**Figures 4J-L**). Furthermore, we tried to investigate the relationship between PLAC8 and immune checkpoints. Interestingly, a robust positive correlation was detected, implying that PLAC8 may hold predictive value for the immunotherapy response in ccRCC (**Figures 4M-P**). Meanwhile, we found that PLAC8 was mainly expressed in the mono/macro and NK cell at single-cell level (**Figure S1**).

### Role of PLAC8 in ccRCC genomic features

**Figure 5:**
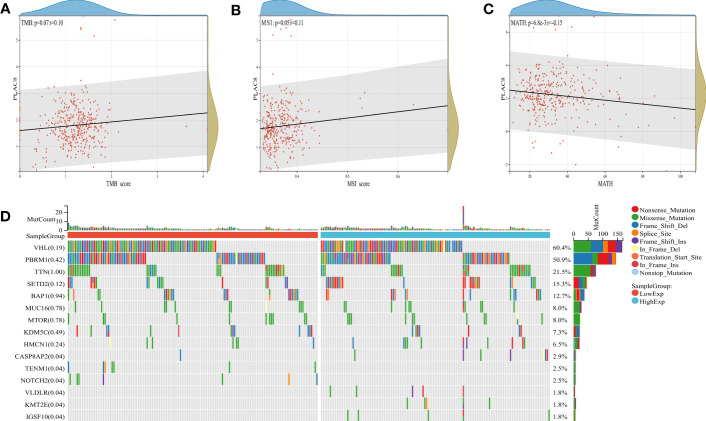
Genomic features of PLAC8. **(A-C)** Correlation of PLAC8 with TMB, MSI and MATH; **(D)** Ranked mutated genes.

A significant association has been observed between tumor mutational burden (TMB) and the efficacy of PD-1/PD-L1 inhibitors. MSI and MATH can reflect genomic instability and tumor heterogeneity. Our findings revealed a positive correlation between PLAC8 expression and TMB as well as MSI, while demonstrating a negative correlation with MATH (**Figures 5A-C**). **Figure 5D** demonstrated the genomic mutation feature of PLAC8 and ranked mutated genes.

### Immunotherapy efficacy and nomogram model of PLAC8 in ccRCC

**Figure 6:**
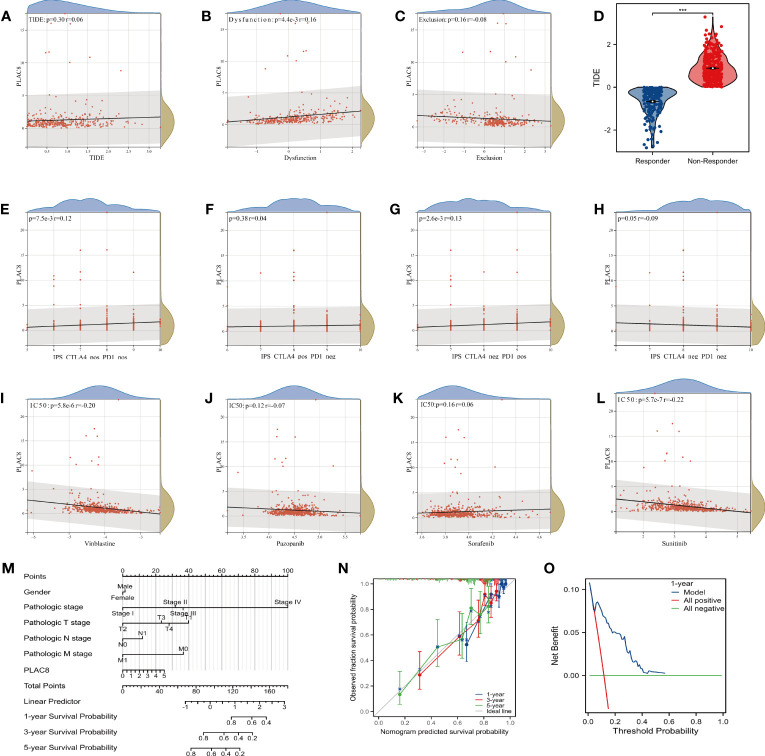
Immunotherapy efficacy and nomogram model of PLAC8. **(A-C)** Correlation of PLAC8 with TIDE, immune dysfunction and exclusion; **(D)** TIDE score in different immunotherapy response groups; **(E-H)** IPS score and PLAC8 expression; **(I-L)** Relation of PLAC8 expression and commonly used immunotherapy or chemotherapy drug sensitivity; **(M)** Nomogram plot constructed on PLAC8 expression and related clinical characteristics; **(N)** The calibration prediction curve; **(O)** The DCA prognosis curve. *** = P < 0.001.

TIDE analysis revealed that PLAC8 seemed to be associated with immune dysfunction yet unrelated to the TIDE score and immune exclusion (**Figures 6A-C**). The TIDE score had a certain predictive effect on immune response (**Figure 6D**). Furthermore, a modest correlation was detected between PLAC8 expression and both IPS CTLA4(+)/PD1(+) and CTLA4(-)/PD1(+) subsets, suggesting the potential of PLAC8 as a predictive marker for immunotherapy response in ccRCC patients (**Figures 6E-H**). Through the analysis of the commonly used target drug sensitivity for ccRCC, we selected Vinblastine and Sunitinib. It seemed that PLAC8 could enhance their drug sensitivity (**Figures 6I-L**). After that, we established a nomogram plot based on PLAC8 expression and related clinical characteristics (**Figure 6M**). The calibration curve we constructed demonstrated a good fit between the predicted model and the actual (**Figure 6N**). DCA curves indicated that our model could increase the prediction performance of PLAC8 in ccRCC prognosis (**Figure 6O**).

### Knockdown of PLAC8 reduced the malignant biological behaviors of ccRCC

**Figure 7:**
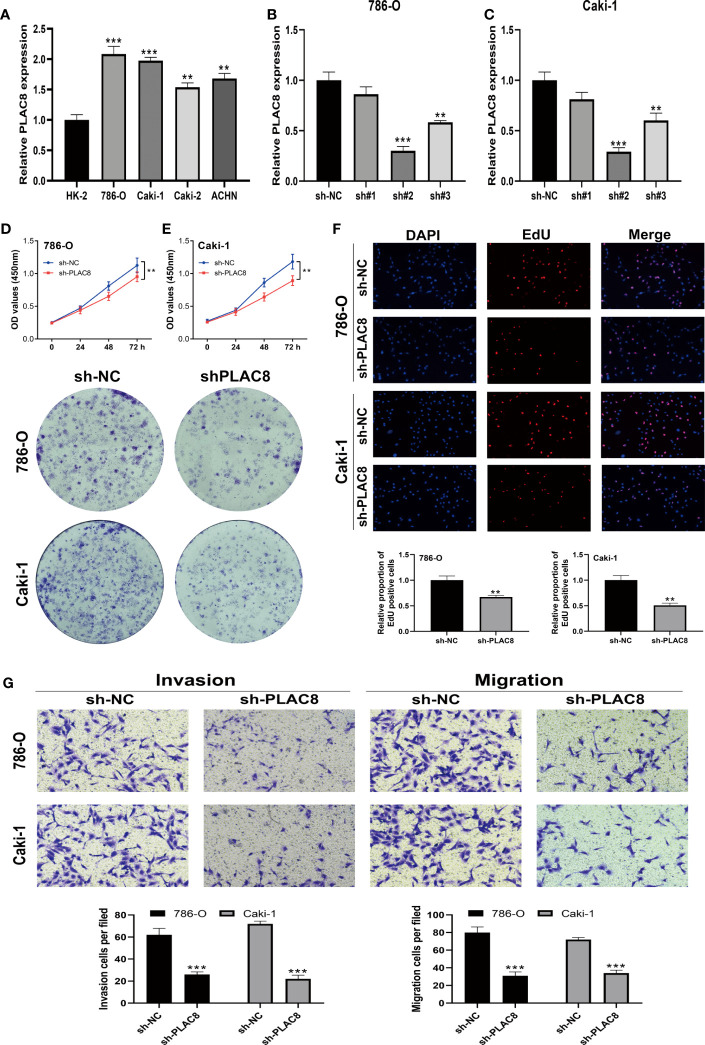
Knockdown of PLAC8 reduced the malignant biological behaviors of ccRCC. **(A)** The expression of PLAC8 in different cell lines; **(B, C)** Knockdown efficiency of the three designed specific shRNAs; **(D–F)** The CCK8 assay, clonogenic assay and the EdU assay; **(G)** Evaluation of PLAC8 on cell invasion and metastasis using Transwell assay, ** = P< 0.01, *** = P< 0.001.

Subsequently, we examined the expression level of PLAC8 in HK-2 and RCC cell lines (786-O, Caki-1, Caki-2, and ACHN). In contrast to the normal kidney cell line HK-2, all RCC cell lines consistently displayed markedly higher expression levels of PLAC8 (**Figure 7A**). Then we selected 786-O and Caki-1 for specific knockdown of PLAC8 to investigate its effect on the biological behavior of RCC cells. We found that among the three designed specific shRNAs, sh#2 had the best knockdown efficiency and was therefore selected for subsequent experiments (**Figures 7B, C**). Both the CCK-8 assay and clonogenic assay consistently revealed a profound inhibition of RCC cell proliferation after the knockdown of PLAC8 in RCC cell lines 786-O and Caki-1 (**Figures 7D, E**). The results of the EdU assay demonstrated that the DNA replication activity of kidney cancer cells was significantly reduced after the knockdown of PLAC8 (**Figure 7F**). Furthermore, we employed the Transwell assay to examine the impact of PLAC8 on cell invasion and metastasis. Remarkably, our results consistently exhibited a pronounced reduction in the invasive and metastatic abilities of RCC cells upon the knockdown of PLAC8 in both 786-O and Caki-1 cell lines (**Figure 7G**).

### PLAC8-derived molecules constructing the prognosis model based on machine learning

**Figure 8:**
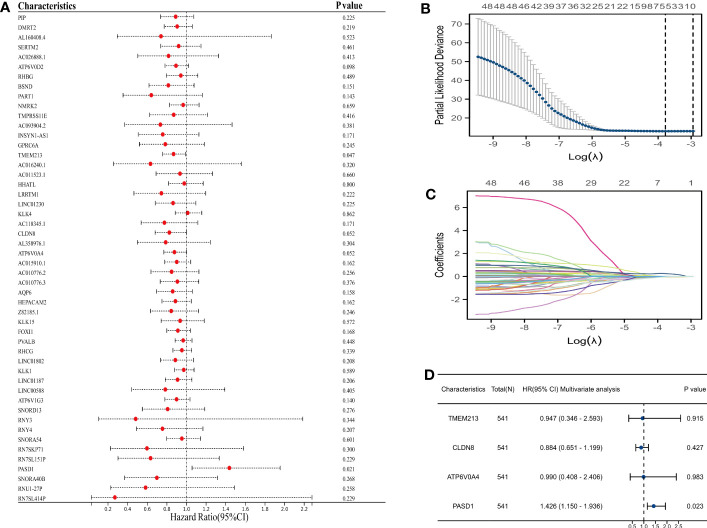
Prognosis model construction based on machine learning. **(A)** The top 50 prognosis-related genes; **(B, C)** LASSO logistics regression to help find the optimal variable; **(D)** Multivariate cox regression analysis.

**Figure 9:**
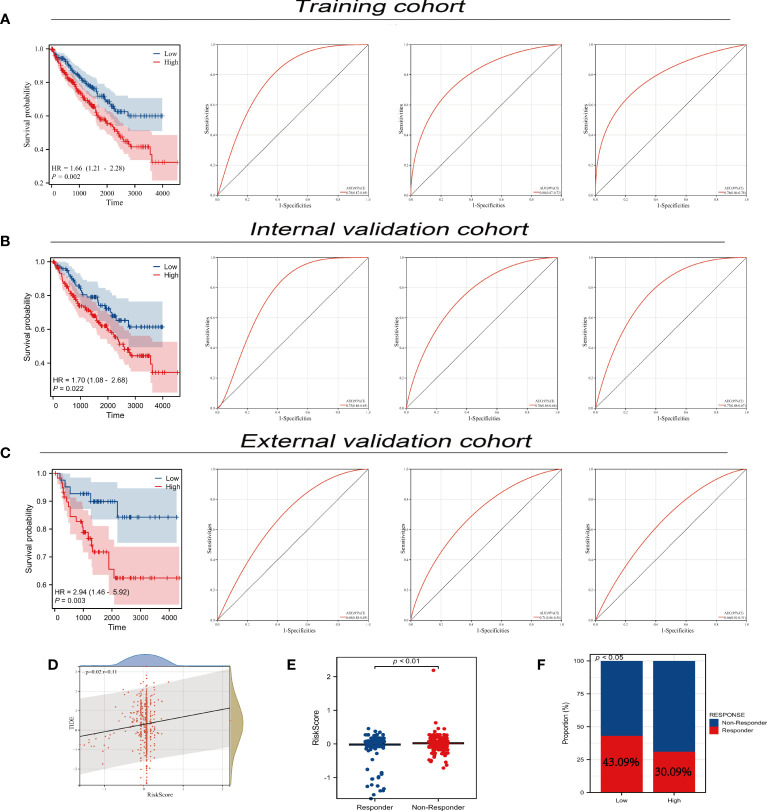
Predictive performance of prognosis model. **(A-C)** Predictive performance of our prognosis model in three different cohort; **(D)** Correlation analysis between TIDE and risk-score; **(E)** The diverse distribution of risks-sore in immunotherapy responders and non-responders; **(F)** The response rate of immunotherapy in different PLAC8 expression groups.

We utilized univariate Cox regression to analyze PLAC8-derived prognosis-related molecules. **Figure 8A** exhibited the top 50 genes. Then we applied LASSO logistics regression to help find the optimal variable (**Figures 8B, C**). A prognosis signature was built successfully based on Cox regression analysis(Risk score=0.0765 + TMEM213 * -0.1546 + CLDN8 * -0.1239 + ATP6V0A4 * -0.0097 + PASD1 * 0.3546) (**Figure 8D**). Subsequently, the predictive performance of the model was assessed in three distinct cohorts. The survival analysis revealed that patients characterized by high-risk scores exhibited a significantly worse OS, further highlighting the excellent predictive capability of our model (**Figure 9A**). Luckily, our model presented a satisfactory performance in the other two cohorts (**Figures 9B, C**). Moreover, our findings revealed a significant positive correlation (R = 0.11, P = 0.02) between the Risk Score and TIDE score, providing further evidence of their interconnectedness (**Figure 9D**). We also noticed that immunotherapy responders tend to get a lower score and the percentage of which was higher in low-risk patients than in the high group (**Figures 9E, F**).

## Discussion

RCC is a prevalent malignancy that originates from the kidney and ranks among the most common malignant tumors worldwide. By 2021, RCC was the thirteenth most common malignancy globally (22). According to statistics, the incidence of RCC in all cancers is about 3%, and its incidence is increasing each year (23). There are three major histological subtypes of RCC, of which the most common subtype is clear cell carcinoma, accounting for 75-80% (24). When ccRCC is diagnosed early, the cure rate is high, and surgery is the primary treatment. A previous study reported that patients with organ-related RCC who underwent surgery achieved an impressive 10-year cancer-specific survival rate ranging from 85% to 96% (25). However, treating advanced diseases is complex and the mortality rate is high. The mortality rate of RCC is 30%-40% (1). Finding new biomarkers for the diagnosis and treatment of ccRCC is quite significant.

Our study explored the role of PLAC8 remodeling the immune microenvironment in ccRCC. Through meticulous bioinformatics analyses, we consistently observed a marked increase in PLAC8 expression levels in ccRCC at both the RNA and protein levels. The analysis of clinical features revealed distinct variations in PLAC8 expression concerning the T stage, M stage, and histological grade. Prognostic analysis showed that patients with high PLAC8 expression were likelier to have adverse prognostic outcomes. We also explored the possible role of PLAC8 in developing ccRCC by enrichment analysis of differential genes. The findings from immune infiltration analyses and multiple immune cell analyses provided evidence that PLAC8 participates in modulating the immune microenvironment of ccRCC and consequently influences the efficacy of immunotherapy. *In vitro* cell experiments showed that the knockdown of PLAC8 resulted in a decreased proliferation, invasion and migration ability of RCC cells. Finally, the prognostic model constructed by PLAC8-derived molecules has a strong predictive ability for the prognosis of patients.

Bioconcentration analysis showed that the enrichment was found in cell cycle checkpoints, mitotic G1 phase and G1/S transition, DNA replication, and cell cycle mitotic enrichment. The cell cycle is essential in controlling cell proliferation and tumor growth. Hwang et al. revealed that Sunitinib has an anti-tumor effect. The cell cycle of renal cancer is blocked by Ginsenoside Rh2, which sensitizes the effect of Sunitinib and slows down the tumor progression (26). In the cell cycle, the mitotic G1 phase and G1/S transition checkpoint are important steps. Based on vitro experiments, Zhang et al. Implied human kidney cancer cell lines 786-O and Caki-2 could be arrested in G0/G1 phase by metformin and VPA, blocking tumor proliferation (27). Zou et al. indicated G0/G1 to S transition would be promoted by CCND1, whose expression was negatively correlated with the change of PLAC8 in HCC (28). However, the conclusion was the opposite in other tumors (8, 29–31). DNA replication is the process most prone to change and carcinogenesis. Any condition that causes high levels of DNA damage can also trigger replication stress, which is one of the sources of genomic instability and a major marker of pre-cancerous and cancerous cells. Kanu et al. found that replication fork progression was hindered because of MCM7 and DNA polymerase δ reduction. This process was associated with SETD2 depletion in ccRCC cells, which also played a role in the suppression of replication stress (32). Our results suggest that PLAC8 may promote the progression of ccRCC through its impact on the activity of the aforementioned pathways.

According to a variety of immune cell analysis algorithms, our study found PLAC8 could increase CD8+ T cells, macrophages, cytotoxic lymphocytes, NK cells, and monocytic lineage and reduce Th17 cells in the microenvironment of ccRCC. Contrary to other solid tumors, CD8+ T cells’ infiltration has a connection with poor clinical outcomes in RCC (33–35). The research conducted by Wu et al. revealed that CD8+ T cells exert notable effects on multiple pathways, including chemokine signaling, cytotoxicity mediated by NK cells and cytokine-cytokine receptor interaction. An increased abundance of CD8+ T cells in a state of T cell exhaustion was observed within the tumor microenvironment (TME) of ccRCC, consequently impacting prognosis and immunoevasive outcomes (36). Besides, the role of cd8+T in ccRCC differs from that in the majority of cancers (37). Dai et al. indicated that CD8+T cells’ immune function was reduced with CXCL13+CD8+T cells abundance, which was related to immunoevasive contexture (38). Macrophages release pro-inflammatory cytokines to participate in the creation of a microenvironment that promotes gene mutation production, which leads to tumorigenesis. Fu et al. macrophages enhanced the function of Tregs in glutamine-addicted ccRCC by secreting IL-23 (39). Braun et al. hypothesized that terminally exhausted M2-like macrophages and CD8+T cells inhibit each other, forming an immune dysfunction circuit. This may result in a worse prognosis by inhibiting anti-tumor immune activity in advanced disease (40). Geissler et al. revealed that a higher proportion of tumor-infiltrating NK cells tend to get a higher survival rate in RCC (41). In prostate cancer, Miyahara et al. considered Th17 cells to mediate an anti-tumor effect since the negative correction between infiltration of Th17 cells into tumors and the Gleason score (42). These results indicate that PLAC8 participates in reshaping the immune microenvironment, consequently influencing cancer development through intricate biological interactions.

Our study focused on the construction of a diverse range of prognostic models through the implementation of machine learning algorithms. Following careful evaluation, we identified an optimal model that exhibited exceptional fitting performance. These models point out that patients with high-risk scores tend to have poorer clinical outcomes, indicating that our model has high clinical predictive value. These conclusions provide a new direction for the immunotherapy of ccRCC.

Our research had limitations as follows: Firstly, most of the samples in our database are from Westerners, so our data are more targeted at whites and blacks, which makes the universality of the research need to be further explored. To ensure the broader applicability of our findings, we should conduct further studies focusing on diversifying the sample population by including individuals from different ethnic backgrounds. Secondly, our experiments are mainly carried out *in vitro*. To fully validate our findings and translate them into clinical applications, it is imperative to conduct further experiments *in vivo* using animal models. Thirdly, while our research has shed some light on the role of PLAC8 in ccRCC’s immune microenvironment, the specific underlying mechanisms remain unclear. To address this knowledge gap, we should delve deeper into the molecular pathways and cellular interactions through which PLAC8 modulates the immune response in ccRCC.

## Data availability statement

The raw data supporting the conclusions of this article will be made available by the authors, without undue reservation.

## Author contributions

XS, ZL, QY and YC performed the R analysis. XS and YC performed the experiments. XS, YS and ZL conducted data download and organization. QZ, JY and RJ designed the whole work. All authors contributed to the article and approved the submitted version.

## Supplementary material

Supplementary Figure 1 Single-cell level of PLAC8 in ccRCC. **(A-L)** The expression level of PLAC8 in ccRCC cohort at single-cell level.
