# Supplementary figures and images for: PLAC8 is an innovative biomarker for immunotherapy participating in remodeling the immune microenvironment of renal clear cell carcinoma
Source: Front Oncol. 2023 Oct 30;13:1207551. doi: 10.3389/fonc.2023.1207551 (PMC10643208; doi:10.3389/fonc.2023.1207551)

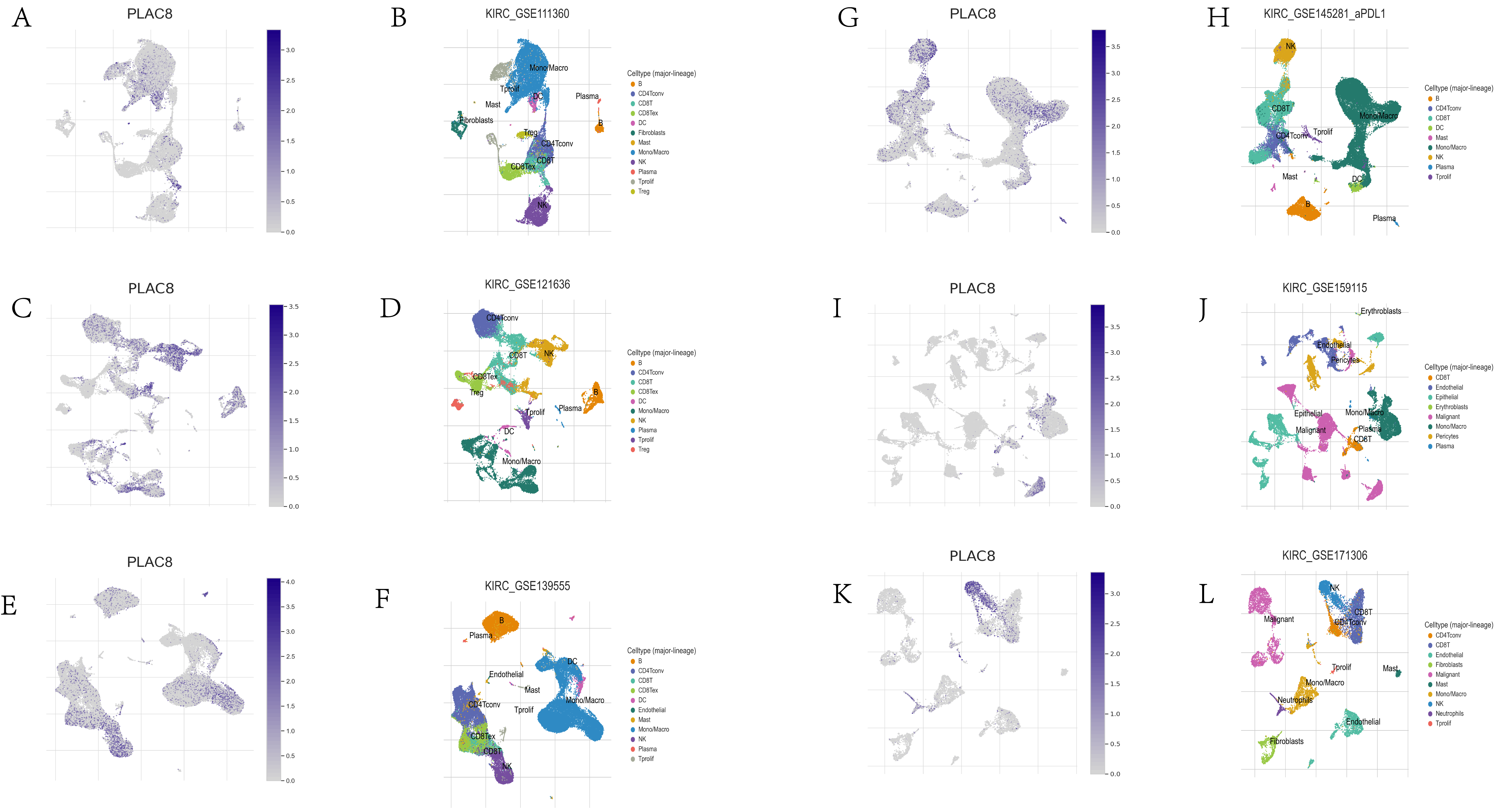

Supplement: Supplementary Figure 1 — Single-cell level of PLAC8 in ccRCC. (A-L) The expression level of PLAC8 in ccRCC cohort at single-cell level. [file Image_1.tif]
